# Azadirachtin Affects the Growth of *Spodoptera litura* Fabricius by Inducing Apoptosis in Larval Midgut

**DOI:** 10.3389/fphys.2018.00137

**Published:** 2018-02-27

**Authors:** Benshui Shu, Jingjing Zhang, Gaofeng Cui, Ranran Sun, Xin Yi, Guohua Zhong

**Affiliations:** Key Laboratory of Crop Integrated Pest Management in South China, Ministry of Agriculture, Key Laboratory of Natural Pesticide and Chemical Biology, Ministry of Education, South China Agricultural University, Guangzhou, China

**Keywords:** *Spodoptera litura*, azadirachtin, midgut, growth regulation, apoptosis

## Abstract

Azadirachtin, the environmentally friendly botanical pesticide, has been used as an antifeedant and pest growth regulator in integrated pest management for decades. It has shown strong biological activity against *Spodoptera litura*, but the mechanism of toxicity remains unclear. The present study showed that azadirachtin inhibited the growth of *S. litura* larvae, which was resulted by structure destroy and size inhibition of the midgut. Digital gene expression (DGE) analysis of midgut suggested that azadirachtin regulated the transcriptional level of multiple unigenes involved in mitogen-activated protein kinase (MAPK) and calcium apoptotic signaling pathways. Simultaneously, the expression patterns of some differentially expressed unigenes were verified by quantitative real time-PCR (qRT-PCR). In addition, the enhanced terminal deoxynucleotidyl transferase biotin-dUTP nick end labeling (TUNEL) staining, the increased expression of caspase family members and apoptosis-binding motif 1 (IBM1) on both gene and protein level and the release of cytochrome c from mitochondria to cytoplasm were induced in midgut after azadirachtin treatment. These results demonstrated that azadirachtin induced structural alteration in *S. litura* larval midgut by apoptosis activation. These alterations may affect the digestion and absorption of nutrients and eventually lead to the growth inhibition of larvae.

## Introduction

The use of synthetic, chemical pesticides is the most effective method for agricultural pest control. However, the conventional pesticides could induce pest resistance (Tilman et al., 2002; Duhan et al., 2017). And the residues of pesticides also confer negative consequences on human health and environment (Cox and Surgan, 2006). Combined, these issues have created an urgent need for sustainable, effective pest control solutions (Helps et al., 2017; Moshi and Matoju, 2017; Patil et al., 2017). The botanical pesticides could be considered to be an alternative tool for integrated pest management due to the advantages of rapid degradation in environment, low toxicity to mammals, and a low risk of resistance development in target pest populations (Schmutterer, 1990; Greenberg et al., 2005; Ahmad et al., 2013).

Currently, the most promising, effective botanical insecticide for integrated pest management is azadirachtin, a tetranortriterpenoid extracted from *Azadirachta indica* (*A. Juss*). It has been widely used for decades and affected three aspects of insect biology: feeding behavior, growth, and development (Rembold et al., 1982; Isman et al., 1990; Wang et al., 2015). For example, azadirachtin exhibited a significant antifeedant effect on *Drosophila melanogaster, Plutella xylostella*, and *Galleria mellonella* (Sezer and Ozalp, 2011; Huang et al., 2016; Kilani-Morakchi et al., 2017). The antifeedant effect was mediated via the activation of deterrent receptor situated in the medial sensillum styloconicum (Liner et al., 1995). It could also inhibit the transmission of cholinergic nerve signaling and calcium channel in suboesophageal ganglion and reduce the frequency of miniature excitatory postsynaptic currents (mEPSCs). These reductions in nerve cell conductivity resulted in the dysfunction of pest central nervous system and promoting antifeedant behavior (Qiao et al., 2014). In addition to feeding behavior, azadirachtin also inhibited insect growth and development. In *D. melanogaster*, azadirachtin inhibited the development via blocking the biosynthesis of ecdysteroids and juvenile hormones (Lynn et al., 2012; Lai et al., 2014). It also blocked the release of prothoracicotropic hormone (PTTH) from neuroendocrine cells, which inhibited the development and molting of *Trypanosoma cruzi* (Garcia et al., 1990; Cortez et al., 2012). Besides, azadirachtin disrupted the proper functioning of endocrine and neuroendocrine systems in *Labidura riparia* (Sayah et al., 1998).

The tobacco cutworm, *S. litura* Fabricius (Lepidoptera: Noctuidae), is a polyphagous pest that feeds on more than 150 different host plants and widely distributed throughout tropical and subtropical regions (Gong et al., 2014; Selin-Rani et al., 2016). It has caused devastating destruction to many important field crops and vegetables, such as cotton, soybeans, and cabbage (Kaur et al., 2016; Kiran Gandhi et al., 2016). Azadirachtin has shown significant antifeedant and growth inhibitory action on *S. litura* at the concentration of 10–100 parts per million (ppm) and 1–10 ppm respectively (Govindachari et al., 1996). In addition, the fecundity was also reduced significantly (Nathan and Kalaivani, 2006). It was also demonstrated that azadirachtin had other significant physiological effects on *S. litura*, including deformity of larvae, pupae and adult, reduction of the protein synthesis of pupae, hemolymph volume of the last instar larvae, enzyme activities of larvae gut, and cuticular protein level changes of larvae (Sharma et al., 2003; Huang et al., 2004; Nathan et al., 2005; Jeyasankar et al., 2011; Yooboon et al., 2015). Furthermore, the plasma membrane damage and organelle degeneration in plasmatocytes and granular haemocytes were also induced by azadirachtin (Sharma et al., 2003).

The insect midgut functions as the important site for food digestion and nutritional absorption during insect growth and development. It was reported that azadirachtin could exert multiple effects on pest midgut. For example, azadirachtin reduced the levels and activities of midgut digestive enzymes of *Glyphodes pyloalis* (Khosravi and Sendi, 2013). Additionally, azadirachtin disturbed the serotoninergic system of stomatogastric ganglia and inhibited the peristalsis of midgut in *Locusta migratoria* (Trumm and Dorn, 2000). However, the mechanism of azadirachtin exposure to the midgut of *S. litura* has not been clearly defined. Recent study has found that azadirachtin induced apoptosis in SL-1 cell line, which was derived from the ovary of *S. litura* (Huang et al., 2011). More recently, autophagy-related gene 5 was confirmed to be the molecular switch of autophagy and apoptosis induced by azadirachtin in SL-1 cells (Shao et al., 2016). Therefore, we speculated that the mechanism of azadirachtin could be related to apoptosis *in vivo*.

In order to elucidate the adverse effects of azadirachtin on *S. litura* larvae, the present study investigated the morphology and histopathological changes of larvae after azadirachtin expose. Digital gene expression (DGE) analysis of midgut showed that multiple apoptotic signaling pathways involved in the process. In addition, qRT-PCR, TUNEL and western blot had been accomplished and further confirmed that azadirachtin induced apoptosis in the midgut of *S. litura* larvae. Our data could provide further insight into the mechanism of azadirachtin for growth regulation and it might benefit for the application of these efficient botanic pesticides.

## Materials and methods

### Reagents and antibodies

Azadirachtin (95% purity, #A7430, Sigma-Aldrich, St. Louis, MO, USA) was dissolved into dimethyl sulfoxide (DMSO, #D103272, Aladdin, Shanghai, China) and mixed with artificial diet. The antibody of Sl-IBM1 was prepared by our laboratory. Cleaved Caspase-3 (Asp 175) antibody was obtained from Cell Signaling Technology (#9661, USA) and mouse polyclonal anti-cytochrome c was purchased from Beyotime Biotechnology (#AC909, Shanghai, China). Other chemicals were domestic products.

### Insect culture and treatment

The *S. litura* larvae maintained in our laboratory (Guangzhou, China) were fed with an artificial diet upon hatching and maintained in a stable condition of 25 ± 1°C, 60–70% relative humidity and a 16:8 h light: dark cycle (Yi et al., 2017). The adults were maintained with 10% honey water. The third-instar larvae fed with the artificial diet supplemented with 1 μg/g azadirachtin for 7 d were used for the following experiments and defined as larvae with azadirachtin treatment. The larvae fed with the artificial diet containing 200 μL/kg DMSO were used as the control group.

### Hematoxylin–eosin staining

Azadirachtin-treated and control larvae were dissected and the midgut was washed in cold phosphate buffered saline (PBS) and fixed with 4% paraformaldehyde (#G1101, Servicebio, Wuhan, China) at 4°C for more than 24 h. Then it was embedded in paraffin wax, sectioned at 4 μm slices, mounted on glass slides and stained by hematoxylin and eosin. Slides were visualized on a microscope (Nikon, Japan).

### cDNA library preparation and illumina sequencing

Total RNA was extracted from the midgut samples using TRIzol (#15596026, ThermoFisher Scientific, USA) as described in the manufacturer's instructions. Purity of total RNA was measured by a NanoDrop® spectrophotometer (Thermo Fihser, MA, USA). RNA integrity was determined by an Agilent 2100 (Agilent Technologies, CA, USA). Two libraries were constructed, one for the midgut of azadirachtin-treated larvae and one for the control. A total of 1 μg qualified RNA per sample was used for the library preparation. The sequencing libraries were generated by VAHTS mRNA-seq v2 Library Prep Kit for Illumina® (Catalog NR601, Vazyme, Nanjing, China) following manufacturer's protocol. The clustering of the index-coded samples was performed on a cBot Cluster Generation System (Illumia, USA) according to the manufacturer's instructions. After cluster generation, the library preparations were sequenced on an Illumina Hiseq X Ten platform with 150 bp paired-end module. The *de novo* assembly of clean reads was performed by Trinity (settings: –min_contig_length150 –CPU 8 –min_kmer_cov 3 –min_glue 3 –bfly_opts ‘-V 5 –edge-thr = 0.1 –stderr’) (Grabherr et al., 2011). The expression levels of unigenes based on the number of reads uniquely mapped were normalized by Reads Per Kilobase of exon model per Million mapped reads (FPKM) method. The thresholds of differentially expressed unigene were False discovery rate (FDR) ≤ 0.001 and |log_2_Fold change| ≥1. Differentially expressed unigenes were subjected to Gene Ontology (GO) and Kyoto Encyclopedia of Genes and Genomes (KEGG) pathway enrichment analysis.

### Quantitative real time PCR (qRT-PCR)

The total RNA of midgut samples was extracted by TRIzol reagent as above and reverse transcribed into cDNA with the PrimeScript™ RT reagent Kit (#RR047A, TaKaRa, Japan) following the manufacturer's recommendations. qRT-PCR was performed using the iTaq™ Universal SYBR® Green Supermix (#1725271, BIO-RAD, USA) with a CFX Connect™ Real-Time System (BIO-RAD, USA) under the following thermal program: one cycle at 95°C for 3 min, 40 cycles at 95°C for 10 s; 60°C for 10 s; 72°C for 15 s, and one step from 65 to 95°C for the dissociation stage. The primers used for qRT-PCR were listed in Supplement Table 1. Gene expression levels were calculated by 2^−ΔΔCt^ method (Livak and Schmittgen, 2001) and glyceraldehyde 3-phosphate dehydrogenase (GAPDH) was used as the reference gene (Lu et al., 2013).

### Terminal deoxynucleotidyl transferase biotin-dUTP nick end labeling (TUNEL) assay

TUNEL assay is one of the most effective methods for apoptosis detection in tissues (Sarkissian et al., 2014). Paraffin embedded midgut tissue section was prepared as described above. One Step TUNEL Apoptosis Assay Kit (#C1086, Beyotime, Shanghai, China) was used according to the manufacturer's protocol. Briefly, sections were dewaxed in xylene twice for 5 min, fixed with 100% ethanol for 5 min, then dehydrated with 2 min washes in 90% ethanol, 70% ethanol, distilled water. After that, the sections were treated with 20 μg/mL protease K (#1074, Servicebio, Wuhan, China) at 37°C for 30 min and washed three times with PBS. Slides were incubated with the TUNEL reagent in the dark at 37°C for 1 h, washed three times with PBS and counterstained with 4′, 6-diamidino-2-phenylindole (DAPI) prior to mounting in X mounting media. Slides were visualized on a fluorescence microscope (Nikon, Japan).

### Western blot

Whole protein extraction from midgut samples were carried out with the total protein extraction kit (#W034, Nanjing Jiancheng, China) according to the protocol. Cytoplasmic and mitochondrial proteins were extracted using the mitochondrial protein extraction kit (#KGP850, KeyGEN BioTECH, China). Samples were separated on a 12% sodium dodecyl sulfate polyacrylamide gel electrophoresis (SDS–PAGE) gel and transferred to a polyvinylidene fluoride (PVDF) (#IPVH00010, Millipore, USA) with the wet/semi-dry transfer system. The membranes were blocked in tris-buffered saline (TBS: 100 mM Tris–HCl, pH 7.5, 0.9% NaCl) with 5% fat-free milk at room temperature for 2 h and then incubated in primary antibodies (diluted 1:2000 in TBS) at 4°C overnight, washed by TBST (TBS containing 0.1% Tween-20) three times for 5 min and incubated in secondary antibody (1:2000 in TBS) at room temperature for 2 h. Membranes were developed in enhanced chemiluminescence (ECL) reagent (#1705062S, Bio-RAD, USA) and bands were visualized using ECL detection system (Bio-RAD, USA). GAPDH was used as the reference protein to normalize the differences in protein loading.

### Caspase-3 activity assay

Approximately 3–10 mg of midgut samples dissected from larvae were prepared. The protein concentrations were determined with Bradford method. The caspase-3 activity was detected by the Caspase-3 Activity Assay Kit (#C1115, Beyotime, Shanghai, China) according to the manufacturer's protocol. Enzyme activities were measured by the absorbance of samples at 405 nm with a microplate reader (ThermoFisher Scietific, USA).

### Statistical analysis

Data collected were expressed as the mean ± standard deviation. Statistical significance between control and treatment was determined with SPSS 20.0 using Student's *t*-test.

## Results

### Inhibitory effect of azadirachtin on the growth of *S. litura* larvae

**Figure 1:**
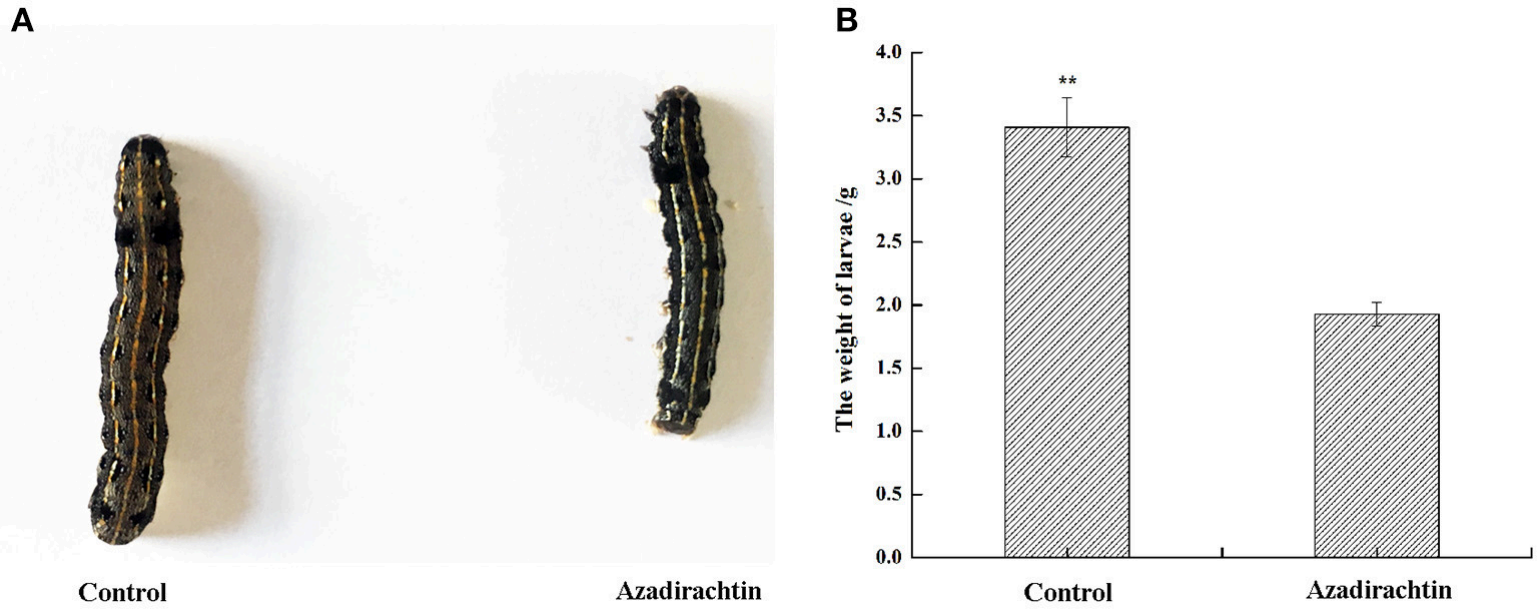
Morphological and dietary changes of *S. litura* larvae with azadirachtin treatment for 7 days. Control: larvae fed with a DMSO added diet; Azadirachtin: larvae fed with azadirachtin added diet. **(A)** Morphological change of larvae fed with azadirachtin added diet; **(B)** dietary change of larvae fed with azadirachtin added diet.

To confirm the growth inhibition phenotype conferred by azadirachtin, third instar larvae were exposed to azadirachtin added or control diet. After 7 days, the larvae fed with azadirachtin exhibited a growth inhibition phenotype. Specifically, the size of larvae with azadirachtin treatment was obviously smaller (Figure 1A). Additionally, the larvae fed with azadirachtin weighed significantly less, with a 43.4% decrease (1.93 g ± 0.093 vs. 3.41 g ± 0.234, *P* < 0.05, *n* = 20) (Figure 1B).

### Morphology and histopathological examination of midgut after azadirachtin treatment

**Figure 2:**
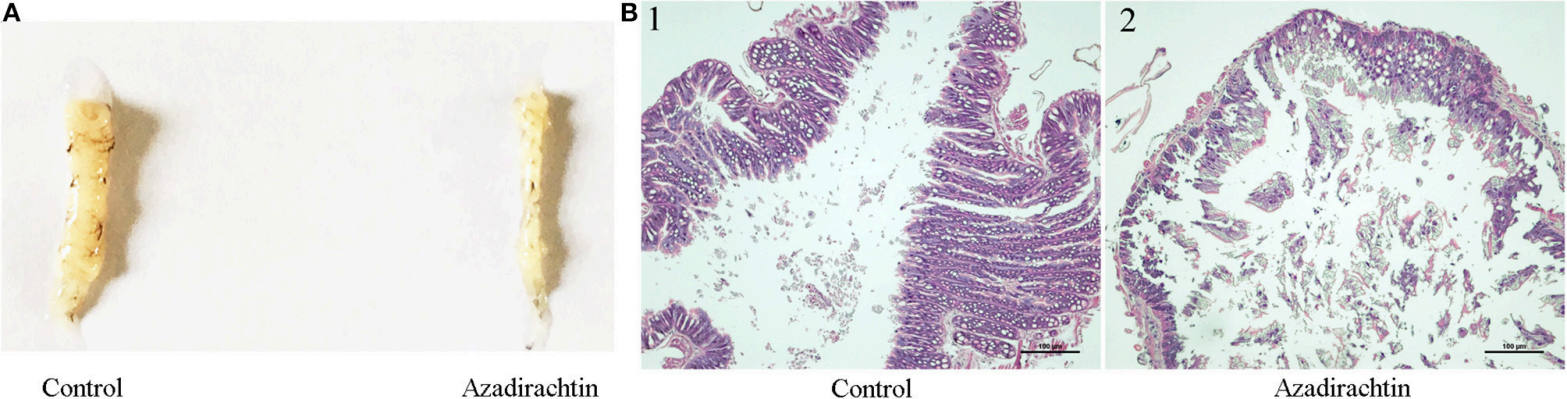
Morphological change and histopathological observation of midgut in *S. litura* larvae fed with azadirachtin added diet. **(A)** Morphological change of midgut in larvae fed with azadirachtin added diet; **(B)** Histopathological observation of midgut in larvae fed with azadirachtin added diet. **(B1)** Control: control group with the normal condition; **(B2)** Azadirachtin: azadirachtin-treated group with significant histopathological change. Scale bar = 100 μm.

In order to determine the effect of azadirachtin on structure, the midgut samples of larvae were dissected, measured and stained with hematoxylin-eosin. After azadirachtin exposure, the size of larval midgut was significantly smaller (Figure 2A). In addition to gross structural changes, azadirachtin also conferred histopathological changes. The midgut of control displayed appropriate, tightly arranged cells (Figure 2B). However, it exhibited cells death, abnormal cell structure, and intestinal wall cracking after azadirachtin treatment (Figure 2B). These data demonstrated that azadirachtin disrupted the normal cell structure and induced cell death in the midgut.

### Differentially expressed genes detected by digital gene expression (DGE) analysis

**Figure 3:**
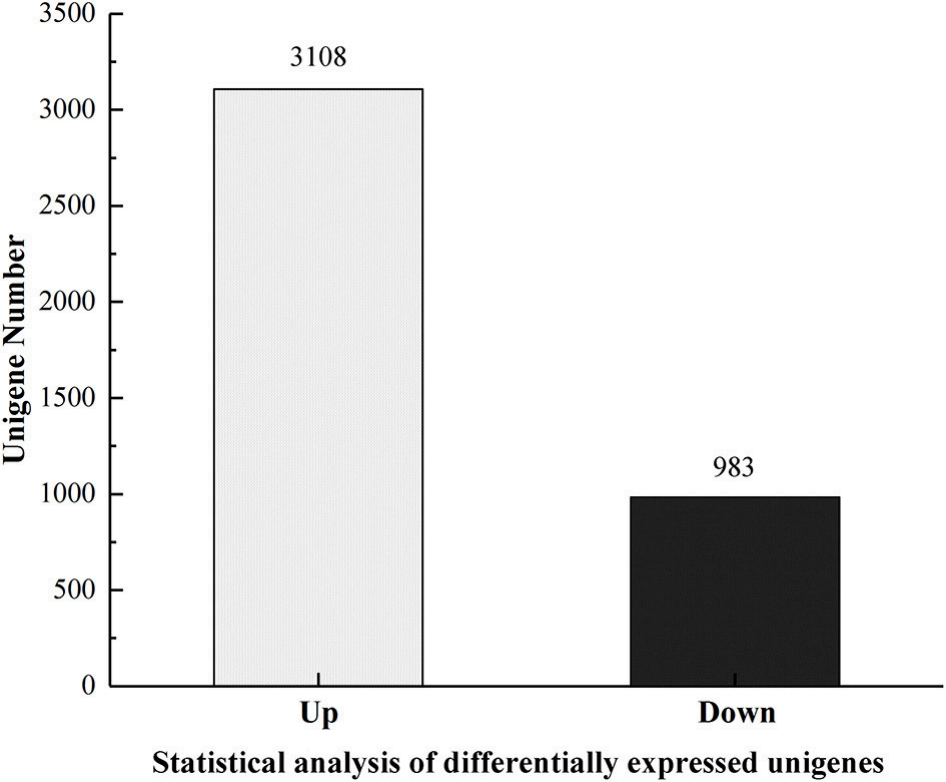
The statistics of differentially expressed unigenes.

In order to uncover the molecular mechanisms underlying the histological changes in the midgut of azadirachtin-treated larvae, transcriptional differences were measured by DGE profiling. As shown in Figure 3, a total of 4,091 unigenes were differentially expressed in midgut of azadirachtin treatment. Of these unigenes, 3,108 were up-regulated and 983 were down-regulated.

The GO enrichment analysis indicated that the differentially expressed unigenes (DEGs) affected by azadirachtin were classified into three categories and 57 functional groups. Among which 1,181 DEGs were assigned into the category of biological process, 915 DEGs to cellular component and 1,177 DEGs to molecular function (Supplement Figure 1). In biological process category, the DEGs assigned in were divided into 24 functional groups and single-organism process was the biggest group with 881 genes assigned, followed by the group of cellular process (881 DEGs), and metabolic process (740 DEGs). As to cellular component category, the biggest group were cell and cell part, which has the same number of 629 unigenes assigned. The DEGs classified into molecular function category were divided into 16 groups and catalytic activity group has the higher percentage of unigenes and 723 unigenes were concentrated.

The DGE analysis revealed 2,154 differentially expressed unigenes were involved in 247 KEGG pathways. Of these pathways, three pathways with the largest number of annotations were metabolic pathways (358 unigenes, 16.62%), pancreatic secretion (181 unigenes, 8.4%), and protein digestion and absorption (148 unigenes, 6.87%) (Supplement Figure 2).

### qRT-PCR validation

**Figure 4:**
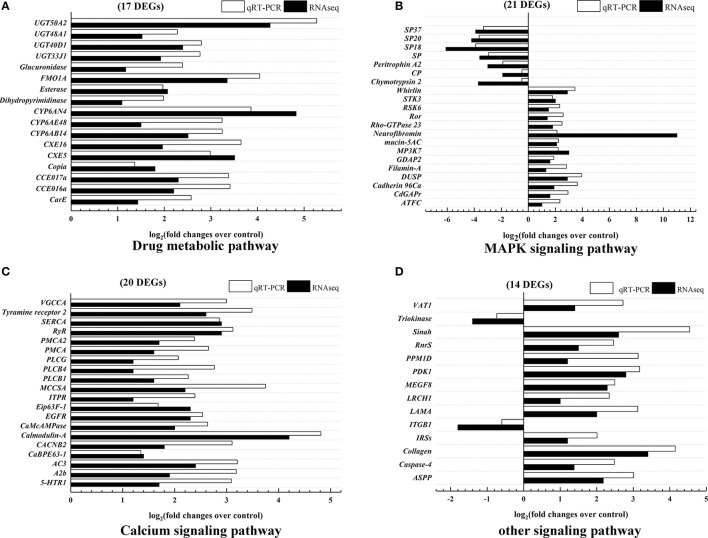
Validation of differentially expressed unigenes expression levels by qRT-PCR. Data were expressed as arithmetic mean of three replications. **(A)** 17 differentially expression genes annotated into drug metabolic pathways. **(B)** 21 differentially expressed genes annotated into MAPK signaling pathway. **(C)** 20 differentially expressed genes in calcium signaling pathway. **(D)** 14 differentially expressed genes in other signaling pathway. All data were normalized by the expression level of GAPDH.

To verify the results of DGE analysis, parts of unigenes with at least a two-fold difference in expression were selected. Because azadirachtin was considered to be a xenobiotic, 17 differentially expressed unigenes involved in xenobiotics biodegradation and metabolism pathway were validated by qRT-PCR (Table 1) and exhibited the same trends as the DGE profiling results (Figure 4A). These results indicated that three major detoxification enzymes (esterase, UDP-glycosyltransferase and cytochrome P450) were activated in midgut after exposure to azadirachtin.

**Table 1:** The differentially expressed unigenes validated by qRT-PCR.

**KEGG pathway**	**Gene ID**	**Blast Nr**	**Log_2_ ratio**	**Up or down**
Xenobiotics biodegradation and metabolism	Unigene14421_All CL901.Contig1_All Unigene12834_All Unigene13067_All Unigene25183_All Unigene77_All Unigene15497_All CL717.Contig3_All CL1676.Contig2_All CL688.Contig2_All Unigene12989_All Unigene24350_All Unigene17153_All CL521.Contig1_All CL700.Contig2_All Unigene10321_All Unigene2255_All	Acetylcholinesterase-like Carboxyl/choline esterase CCE016a Carboxyl/choline esterase CCE017a Copia protein-like Antennal esterase CXE5 Antennal esterase CXE16 Cytochrome CYP6AB14 Cytochrome CYP6AE48 Cytochrome CYP6AN4 Dihydropyrimidinase-like Esterase Flavin-dependent monooxygenase FMO1A Beta-glucuronidase UDP-glycosyltransferase UGT33J1 UDP-glycosyltransferase UGT40D1 UDP-glycosyltransferase UGT48A1 UDP-glycosyltransferase UGT50A2	1.43 2.20 2.30 1.80 3.51 1.96 2.51 1.50 4.83 1.09 2.07 3.35 1.17 1.92 2.39 1.52 4.27	Up Up Up Up Up Up Up Up Up Up Up Up Up Up Up Up Up
MAPK	Unigene3826_All Unigene4415_All Unigene148_All Unigene6770_All Unigene19651_All CL750.Contig4_All CL1041.Contig3_All Unigene2313_All Unigene24632_All CL778.Contig4_All Unigene12988_All CL751.Contig1_All CL1052.Contig1_All Unigene24520_All Unigene1990_All Unigene3926_All Unigene21160_All Unigene1336_All Unigene22040_All Unigene18409_All CL2064.Contig1_All	Activating transcription factor of chaperone GTPase-activating protein CdGAPr Cadherin 96Ca Dual specificity protein phosphatase 14 Filamin-A GDAP2 homolog Mitogen-activated protein kinase kinase kinase 7 Mucin-5AC Neurofibromin rho GTPase-activating protein 23 Tyrosine-protein kinase transmembrane receptor Ror-like Ribosomal protein S6 kinase Serine/threonine-protein kinase 3-like Whirlin Chymotrypsin-like protein 2 Chymotrypsin-like protein precursor Peritrophin type-A domain protein 2 Serine protease Serine protease 18 Serine protease 20 Serine protease 37	1.00 1.60 1.92 2.91 1.27 1.59 3.03 2.06 11.01 1.81 1.44 1.52 2.02 2.92 −3.73 −1.92 −3.02 −3.60 −6.13 −4.18 −3.88	Up Up Up Up Up Up Up Up Up Up Up Up Up Up Down Down Down Down Down Down Down
Calcium	CL582.Contig2_All Unigene6775_All Unigene24543_All CL1096.Contig2_All Unigene17192_All Unigene25518_All CL433.Contig1_All CL1893.Contig2_All CL1096.Contig1_All Unigene4330_All Unigene53_All Unigene6528_All Unigene10766_All Unigene48_All Unigene11257_All CL2173.Contig1_All Unigene25081_All CL1019.Contig3_All	5-hydroxytryptamine receptor 1-like Adenosine receptor A2b Adenylate cyclase type 3 Calcium-binding protein E63-1 Voltage-dependent L-type calcium channel subunit beta-2 Putative calmodulin-A Ca(2+)/calmodulin-responsive adenylate cyclase-like Putative epidermal growth factor receptor Ecdysone-induced protein 63F 1 Inositol 1,4,5-trisphosphate receptor ITPR Muscle calcium channel subunit alpha-1-like Phospholipase C beta 1 Phospholipase C beta 4 Phospholipase C gamma Plasma membrane calcium ATPase Plasma membrane calcium-transporting ATPase 2 Ryanodien receptor Sarco/endoplasmic reticulum calcium ATPase	1.72 1.89 2.37 1.40 1.80 4.21 2.33 2.26 2.29 1.15 2.19 1.56 1.23 1.25 1.64 1.68 2.94 2.94	Up Up Up Up Up Up Up Up Up Up Up Up Up Up Up Up Up Up
	Unigene10168_All Unigene12882_All	Putative tyramine receptor 2 Voltage-gated calcium channel alpha subunit	2.62 2.07	Up Up
Focal adhesion	CL2727.Contig1_All Unigene10872_All Unigene17569_All Unigene16304_All Unigene12923_All	3-phosphoinositide-dependent protein kinase 1 Multiple epidermal growth factor-like domains protein 8 Laminin subunit alpha Collagen Integrin beta 1	2.79 2.29 2.01 3.43 −1.80	Up Up Up Up Down
P53	Unigene4339_All Unigene131_All Unigene20787_All Unigene5990_All CL1421.Contig2_All	Leucine-rich repeat and calponin homology domain-containing protein 1 Probable E3 ubiquitin-protein ligase sinah Protein phosphatase 1D Ribonucleoside diphosphate reductase smAll subunit Vesicle amine transport protein	1.05 2.64 1.18 1.54 1.45	Up Up Up Up Up
Others	Unigene10824_All Unigene18773_All CL305.Contig1_All Unigene113_All	Apoptosis-stimulating of p53 protein 1 isoform X6 Caspase-4 Insulin receptor substrate 1 Triokinase/FMN cyclase-like	2.18 1.38 1.18 −1.4	Up Up Up Down

In addition, many differentially expressed unigenes were mapped into the pathways involved in apoptosis, such as the mitogen-activated protein kinase (MAPK), calcium, and p53 signaling pathways. A total of 55 unigenes in these apoptotic pathways were also validated by qRT-PCR (Table 1). All the selected unigenes expression profiles were consistent with the results of DGE analysis (Figures 4B–D). These results suggested that azadirachtin activated MAPK, Calcium and other apoptosis signaling pathways within larval midgut.

### Detection of azadirachtin-induced apoptosis in the midgut by TUNEL assay

**Figure 5:**
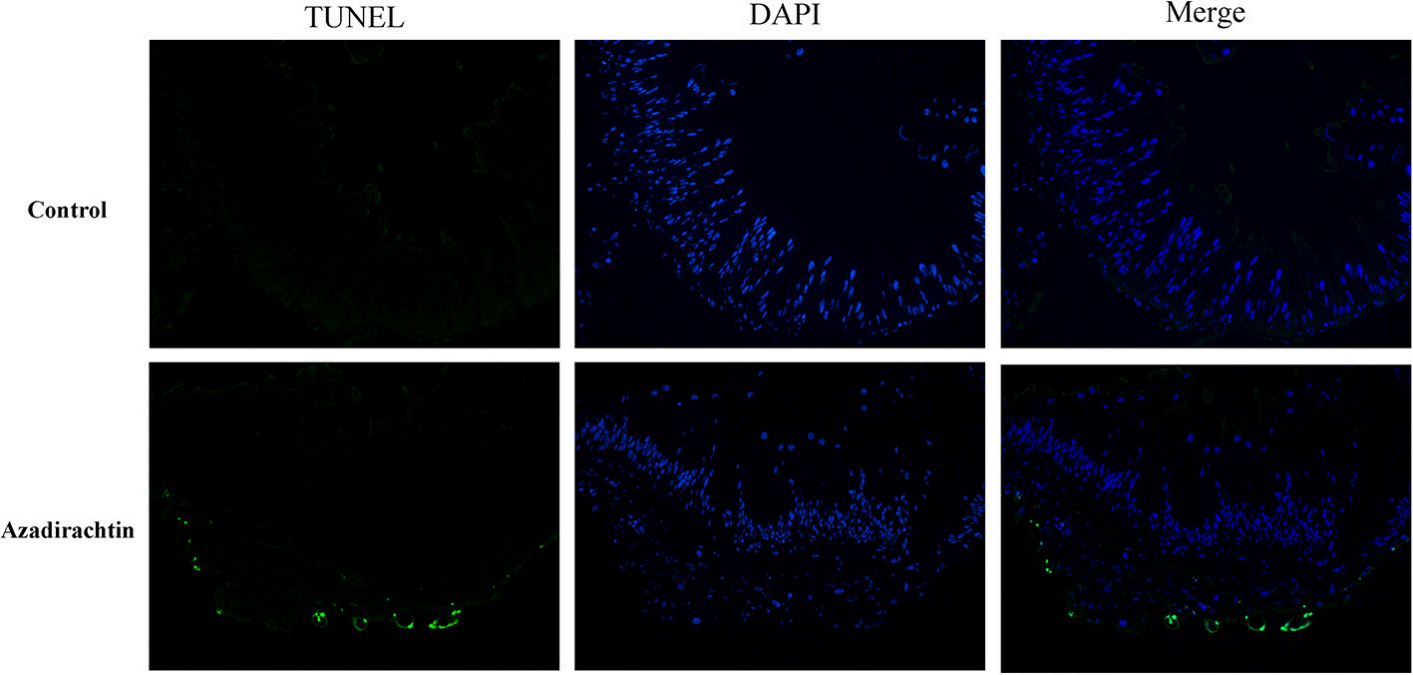
The apoptosis determination by TUNEL assay and DAPI staining in the midgut of *S. litura* larvae fed with azadirachtin added diet. TUNEL-positive cell which was defined as the cell with the green fluorescence. The cell nuclei stained with DAPI was showed with the blue color.

TUNEL assay was utilized to directly demonstrate that azadirachtin induced apoptosis and morphological changes in midgut. The TUNEL-positive cells would fluoresce at 488 nm. TUNEL staining was undetectable in midgut of control larvae, while a high level of TUNEL-positive cells appeared in midgut of azadirachtin exposure (Figure 5). These results indicated that azadirachtin induced apoptosis in midgut.

### Azadirachtin could up-regulate the expression of SL-IBM1

**Figure 6:**
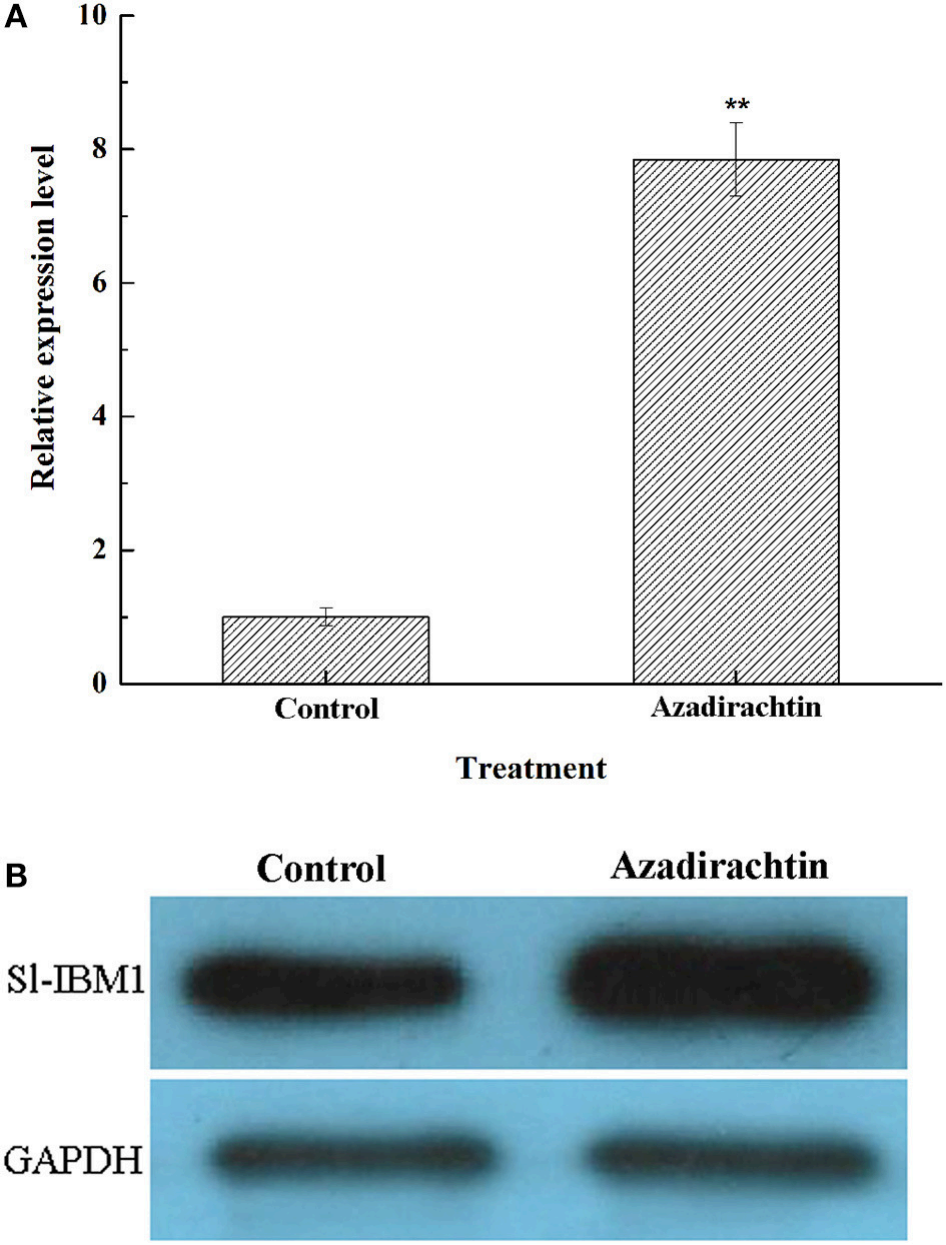
The effects of azadirachtin on mRNA and protein level of IBM1 in larval midgut. **(A)** The mRNA expression change of IBM1 in midgut of larvae fed with azadirachtin added diet. Data were expressed as arithmetic mean ± SEM of three independent experiments. ^*^Significant difference (*P* < 0.05), ^**^significant difference (*P* < 0.01) by ANOVA followed by student's *t*-test. **(B)** The Sl-IBM1 protein level in midgut of larvae fed with azadirachtin and DMSO added diet.

Many unigenes were up-regulated in DGE of azadirachtin-treated midgut. One such differentially expressed unigene IAP-binding motif 1 (*Sl-IBM1*) (log_2_ Ratio = 4.11), an IAP antagonist and Lepidoptera order homolog to *Reaper*, was thought to play an important role in apoptosis process (Wu et al., 2013). Therefore, the expression of IBM1 in midgut after azadirachtin treatment was verified by qRT-PCR and western blot. Azadirachtin exposure caused an up-regulation of *Sl-IBM1* mRNA expression in midgut (Figure 6A). In addition, it could also induce an increase of Sl-IBM1 on protein level (Figure 6B). Taken together, azadirachtin exposure up-regulated the pro-apoptotic factor IBM1.

### Azadirachtin up-regulated the expression of caspases and caspase-3-like activity

**Figure 7:**
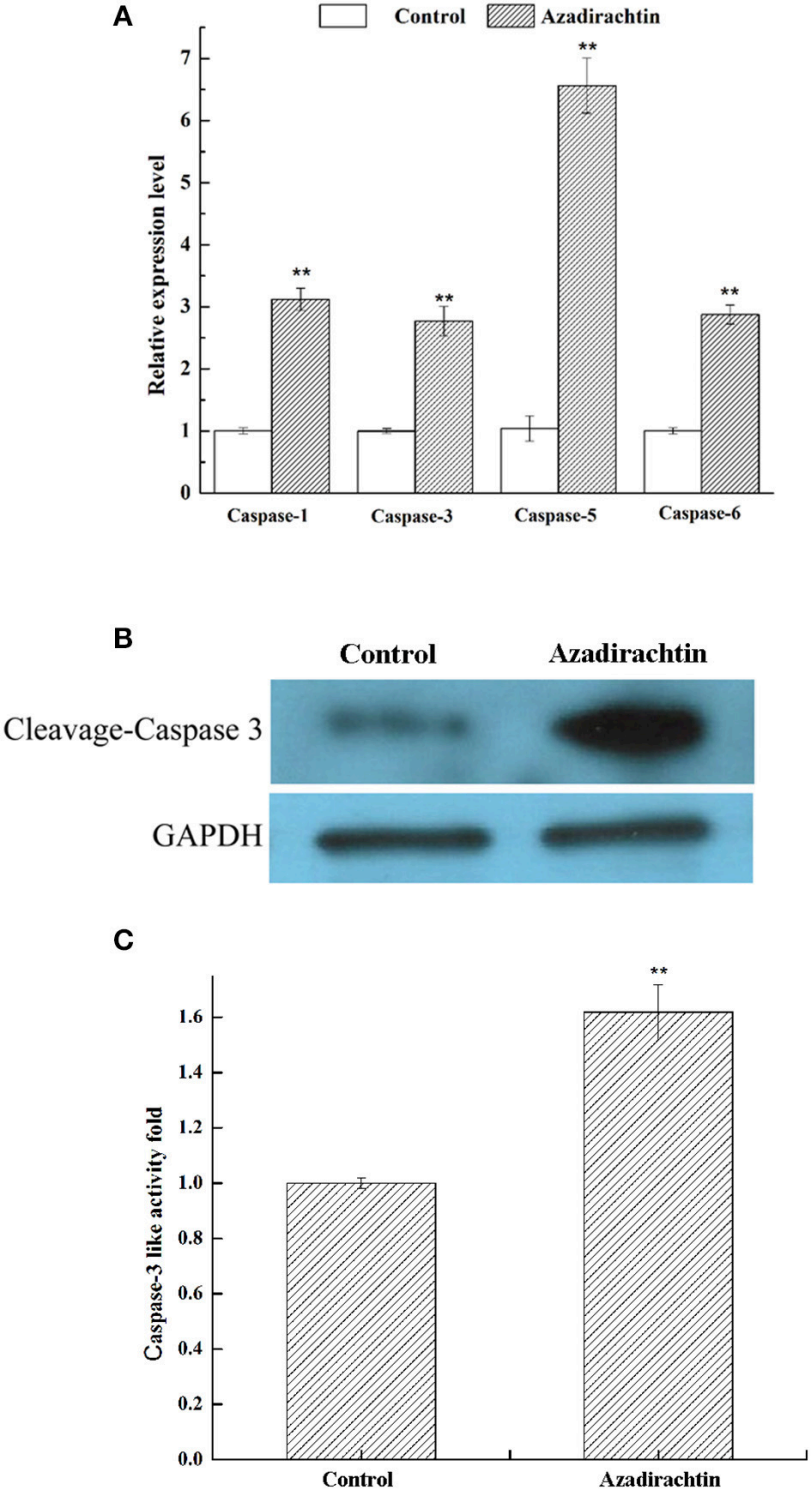
The effects of azadirachtin on mRNA level of caspase family, protein level of cleaved caspase 3 and caspase-3 like activity. **(A)** The mRNA expression changes of caspase family in midgut of larvae fed with azadirachtin added diet. Data were expressed as arithmetic mean ± SEM of three independent experiments. ^*^Significant difference (*P* < 0.05), ^**^significant difference (*P* < 0.01) by ANOVA followed by student's *t*-test. **(B)** The cleaved caspase 3 protein level in midgut of larvae fed with azadirachtin and DMSO added diet. **(C)** The caspase-3 like activity change in midgut of larvae fed with azadirachtin diet.

To determine if azadirachtin exposure and the increased Sl-IBM1 expression induced caspase expression within the midgut, mRNA expression of four caspase genes, including caspase-1, caspase-3, caspase-5, and caspase-6 were compared after azadirachtin exposure by qRT-PCR. All four genes were demonstrated to be up-regulated at least 2.75 folds after azadirachtin treatment (Figure 7A). Simultaneously, the cleaved caspase 3 protein was increased significantly (Figure 7B). Additionally, in accordance with gene and protein expression data, caspase-3-like protease activity was obviously increased (Figure 7C). The up-regulation of caspases expression and caspase-3-like activity confirmed that azadirachtin activated apoptosis in midgut by regulating the caspase-dependent apoptotic pathway.

### Azadirachtin induced the release of cytochrome c in midgut cells

**Figure 8:**
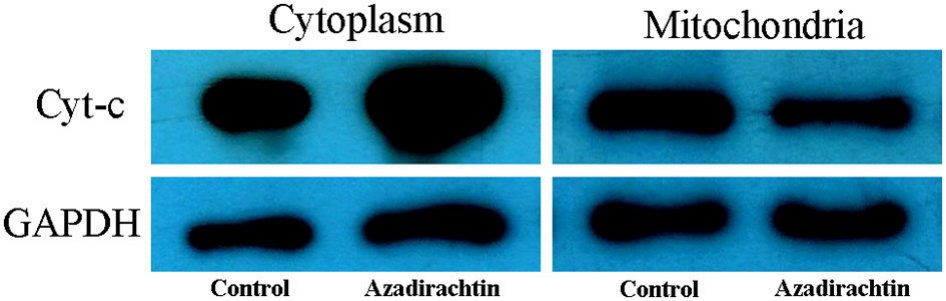
The changes of cytochrome c expression level in cytosolic and mitochondrial of midgut in *S. litura* larvae fed with azadirachtin diet. Control: larvae fed with DMSO added diet; Azadirachtin: larvae fed with azadirachtin added diet.

To reveal whether mitochondria were involved in midgut apoptosis induced by azadirachtin, the protein levels of cytochrome c within cytoplasm and mitochondria of midgut cells were analyzed by western blot. After azadirachtin treatment, cytochrome c in cytoplasmic increased significantly while the mitochondrial reduced (Figure 8). These results indicated that the event of cytochrome c releasing from mitochondria into cytoplasm was occurred in midgut after azadirachtin treatment.

## Discussion

Because of the botanical efficacy, multiple studies have investigated the toxicity of azadirachtin against various insect species. Prior research analyzed the mechanism of the antifeedant activity and regulation of growth *in vitro* and apoptosis induction *in vivo* (Huang et al., 2010; Shu et al., 2015; Xu et al., 2016). Consistent with the prior research, our results demonstrated azadirachtin inhibited the growth of *S. litura* larvae. The insect midgut plays a crucial role in the physiology of growth, including food intake, digestion, and nutrient absorption (Franzetti et al., 2015). It was reported that azadirachtin immediately and severely diminished the conversion of ingested nutrients in *Spodoptera littorali* (Martinez and van Emden, 1999). This conversion was showed to be due, in part, to azadirachtin-induced inhibition of the activity of α-amylase, a digestive enzyme produced by midgut epithelial cells (Rharrabe et al., 2008). Our results revealed that azadirachtin also disrupted the structure of larval midgut epithelium. This destruction likely further disturb the digestion of nutrients, which may contribute to explain the growth regulation mechanisms of azadirachtin in *S. litura*.

Apoptosis is a spontaneous programmed cell death process in response to adverse stimuli (Suganuma et al., 2011; Huang N. et al., 2013). Pesticides could work in part by activating apoptosis. For example, apoptosis occurred as a protective mechanism in midguts, salivary glands and ovaries of honey bee larvae after pesticides treatment (Gregorc and Ellis, 2011). Additionally, apoptosis and autophagy were significantly increased in the brain of worker honey bee (*Apis mellifera*) after exposure to sublethal doses of imidacloprid (Wu et al., 2015). Multiple botanical pesticides also exhibited the same function of apoptosis induction on a variety of insect species. For example, the cry toxins, produced by *Bacillus thuringiensis*, induced mitochondrial permeabilization and led to apoptosis in *Aedes aegypti* larvae (Lemeshko and Orduz, 2013). In addition, previous research of our laboratory has found that camptothecin resulted in midgut epithelial cell apoptosis of *S. litura*, which was correlated with the increased expression of programmed cell death protein 11 (Gong et al., 2014). Our results confirmed that azadirachtin also induced apoptosis in midgut epithelial cells of *S. litura*. These results further supported apoptosis induction in response to pesticide exposure.

Both MAPK and calcium signaling pathways regulated multiple biological processes, including cell proliferation, differentiation and apoptosis (Berridge et al., 2000; Kyriakis and Avruch, 2012; Zhu et al., 2017). Neem limonoids contained azadirachtin have previously been reported to target the MAPK signaling pathway (Nagini, 2014; Sui et al., 2014). Additionally, in *D. melanogaster* S2 cells, azadirachtin induced apoptosis by regulating the Ca^2+^-Calmodulin signaling pathway and mediating the release of intracellular Ca^2+^ (Xu et al., 2016; Humeau et al., 2017). In this study, DGE analysis and qRT-PCR indicated that genes involved in MAPK and calcium signaling pathways were differentially expressed in midgut after azadirachtin exposure. Our data suggested that azadirachtin may also act, in part, through MAPK and calcium signaling pathways to induce apoptosis. Further validation is required to link these pathways to apoptosis activation in response to azadirachtin *in vivo*.

Multiple insect species have been shown to activate apoptosis using homologous pathways in response to negative stimuli. In insects, RHG protein family members and inhibitor of apoptosis (IAP) proteins regulated apoptosis and cell death (Bryant et al., 2009). Additionally, the transcriptional expression of *reaper, hid, grim* preceded apoptosis (Vasudevan and Ryoo, 2015). In *Drosophila*, Reaper induced-apoptosis in response to stimuli required both homodimerization and oligimerization with Hid (Sandu et al., 2010). The oligimerization of Reaper and Hid resulted in the recruitment to mitochondrial membrane and activation of apoptosis (Sandu et al., 2010). Besides, in BmN cells and *Bombyx mori* pupae, the mRNA expression level of *Bm-IBM1* (the Reaper homolog) increased in response to infection with baculovirus *B. mori* Nucleopolyhedrovirus (Wu et al., 2013). In addition, Bm-IBM1 bind to BmIAP1 at the highly conserved IAP-binding motif, which resulted in apoptosis induction (Bryant et al., 2009). Furthermore, Bm-IBM1 induced apoptosis by localizing to mitochondria and activating apoptosis signaling pathway, demonstrating the conservation of Reaper-induced apoptosis across species (Wu et al., 2013). Our data demonstrated that the expression of Sl-IBM1 was also increased in midgut after azadirachtin exposure, which suggested that the transcription level changes of IBM1 in lepidoptera signal apoptosis induction could be one of the indices of apoptosis. Possibly, Sl-IBM1 interacted with IAPs to activate the mitochondrial-associated apoptotic pathways, although no Hid-like protein homolog was found in *S. litura*.

Azadirachtin induced apoptosis in insect cell lines has been studied more detail over past decades. In Sl-1 cell lines, azadirachtin induced the caspase-dependent mitochondrial pathway via activating the cleavage of caspase-3 (Huang et al., 2011). The release of cytochrome c from mitochondria to cytoplasm is the critical step in mitochondrial apoptotic pathway and the cytochrome c released in cytosol bound to apaf-1 and formed an apoptosome, eventually activated caspases and resulted in apoptosis (Liu et al., 2012; Huang J. et al., 2013; Yang et al., 2017). In this study, the increase of caspases mRNA expression, the cleaved caspase 3 protein expression, the up-regulation of caspase-3 like activity and the release of mitochondrial cytochrome c *in vivo* further supported that azadirachtin activated apoptosis via the caspase-dependent mitochondrial apoptotic pathway. Additionally, these results indicated that the mitochondrial apoptotic pathway could be prevalent and plays the critical role in apoptosis activation of lepidoptera insects.

In conclusion, azadirachtin inhibited the growth of *S. litura* larvae by inducing apoptosis and destroying structure of the midgut. DGE analysis suggested that azadirachtin regulated both MAPK and calcium signaling pathways. It also activated the caspase-dependent mitochondrial apoptotic pathway and induced the release of cytochrome c in larval midgut. Our results are the first to demonstrate that azadirachtin caused growth restriction of *S. litura* larvae via apoptosis activation of midgut *in vivo*.

## Author contributions

BS: Conceived and designed the experiments; BS, JZ, GC, and RS: Performance of the experiments; BS and JZ: Analysis of the data; BS: Manuscript writing; XY and GZ: Manuscript editing.

### Conflict of interest statement

The authors declare that the research was conducted in the absence of any commercial or financial relationships that could be construed as a potential conflict of interest.

## Supplementary material

Supplement Figure 1 Gene Ontology (GO) classification of differentially expressed unigenes. Three different classification represent the three basic categories of Go term (The categories from left to right were biological process, cell composition, molecular functions).

Supplement Figure 2 Top 20 pathways enrichment for differentially expressed unigenes. The y-axis indicates the 20 KEGG pathways and the x-axis indicates the richfactor of each KEGG pathway.

Supplement Table 1 Primers used in the paper.
