# Supplementary figures and images for: Azadirachtin Affects the Growth of Spodoptera litura Fabricius by Inducing Apoptosis in Larval Midgut
Source: Front Physiol. 2018 Feb 27;9:137. doi: 10.3389/fphys.2018.00137 (PMC5835231; doi:10.3389/fphys.2018.00137)

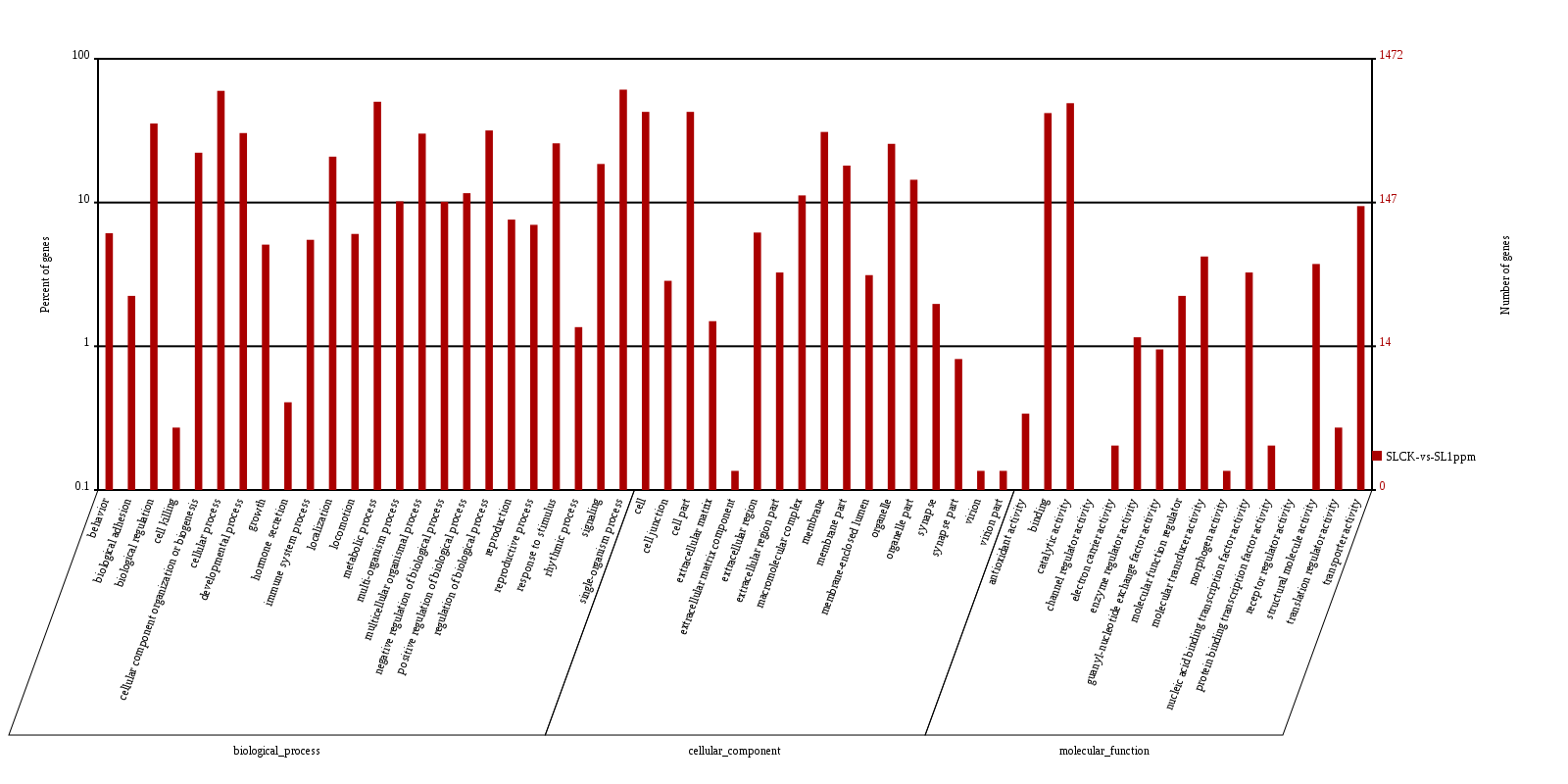

Supplement: Supplement Figure 1 — Gene Ontology (GO) classification of differentially expressed unigenes. Three different classification represent the three basic categories of Go term (The categories from left to right were biological process, cell composition, molecular functions). [file Image1.PNG]

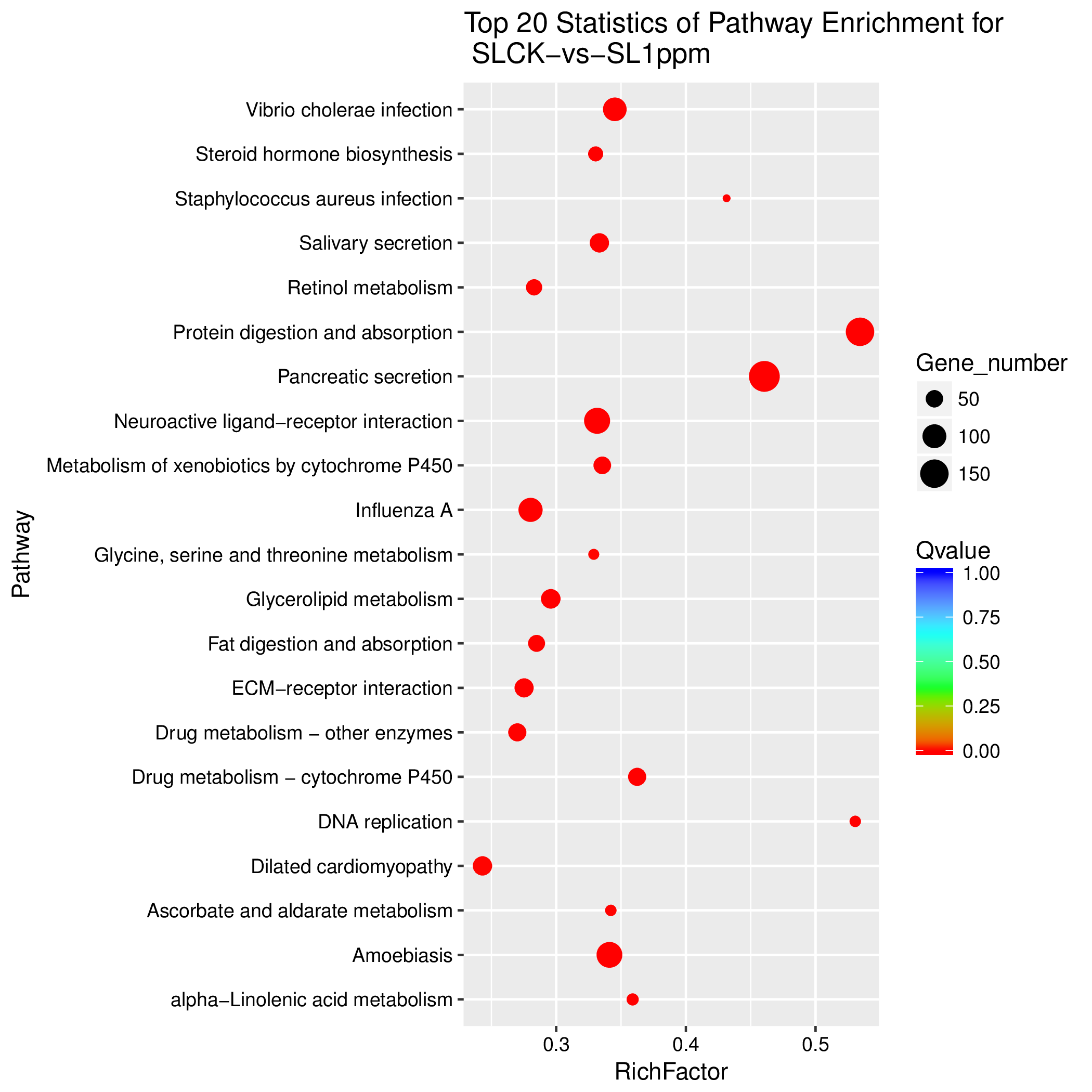

Supplement: Supplement Figure 2 — Top 20 pathways enrichment for differentially expressed unigenes. The y-axis indicates the 20 KEGG pathways and the x-axis indicates the richfactor of each KEGG pathway. [file Image2.PNG]
