# Supplementary material for: Azadirachtin Affects the Growth of Spodoptera litura Fabricius by Inducing Apoptosis in Larval Midgut
Source: Front Physiol. 2018 Feb 27;9:137. doi: 10.3389/fphys.2018.00137 (PMC5835231; doi:10.3389/fphys.2018.00137)
Supplement: Supplement Table 1 — Primers used in the paper. [file Table1.DOCX]

supplement table 1 Primers used in the paper

| Name | **Primers Sequences (5’-3’)** | Name | **Primers Sequences (5’-3’)** |
| --- | --- | --- | --- |
| Sl-IBM1-RT-F:  Sl-IBM1-RT-R  Sl-Caspase-1-RT-F  Sl-Caspase-1-RT-R  Sl-Caspase-3-RT-F  Sl-Caspase-3-RT-R  Sl-Caspase-5-RT-F  Sl-Caspase-5-RT-R  Sl-Caspase-6-RT-F  Sl-Caspase-6-RT-R  Sl-GAPDH-RT-F  Sl-GAPDH-RT-R  Sl-CarE-F  Sl-CarE-R  Sl-CCE016a-F  Sl-CCE016a-R  Sl-CCE017a-RT-F  Sl-CCE017a-RT-R  Sl-Copia-RT-F  Sl-Copia-RT-R  Sl-CXE5-RT-F  Sl-CXE5-RT-R  Sl-CXE16-RT-F  Sl-CXE16-RT-R  Sl-CYP6AB14-RT-F  Sl-CYP6AB14-RT-R  Sl-CYP6AE48-RT-F  Sl-CYP6AE48-RT-R  Sl-CYP6AN4-RT-F  Sl-CYP6AN4-RT-R  Sl-Dihydropyrimidinase-RT-F  Sl-Dihydropyrimidinase-RT-R  Sl-Esterase-RT-F  Sl-Esterase-RT-R  Sl-FMO1A-RT-F  Sl-FMO1A-RT-R  Sl-Glucuronidase-RT-F  Sl-Glucuronidase-RT-R  Sl-UGT33J1-RT-F  Sl-UGT33J1-RT-R  Sl-UGT40D1-RT-F  Sl-UGT40D1-RT-R  Sl-UGT48A1-RT-F  Sl-UGT48A1-RT-R  Sl-UGT50A2-RT-F  Sl-UGT50A2-RT-R  Sl-ATFC-RT-F  Sl-ATFC-RT-R  Sl-CdGAPr-RT-F  Sl-CdGAPr-RT-R  Sl-Cadherin 96Ca-RT-F  Sl-Cadherin 96Ca-RT-R  Sl-DUSP-RT-F  Sl-DUSP-RT-R  Sl-EGF-RT-F  Sl-EGF-RT-R  Sl-Filamin-A-RT-F  Sl-Filamin-A-RT-R  Sl-GDAP2-RT-F  Sl-GDAP2-RT-R  Sl-MP3K7-RT-F  Sl-MP3K7-RT-R  Sl-mucin-5AC-RT-F  Sl-mucin-5AC-RT-R  Sl-Neurofibromin-RT-F  Sl-Neurofibromin-RT-R  Sl-Rho-GTPase 23-RT-F  Sl-Rho-GTPase 23-RT-R  Sl-Ror-RT-F  Sl-Ror-RT-R  Sl-RSK6-RT-F  Sl-RSK6-RT-R  Sl-STK3-RT-F  Sl-STK3-RT-R  Sl-Whirlin-RT-F  Sl-Whirlin-RT-R  Sl-Chymotrypsin 2-RT-F  Sl-Chymotrypsin 2-RT-R  Sl-VAT1-RT-F | AAGAAGTCCAGGAGAATAGGCG  CGAGGGATACCTTTATGATGGC  TAGACAGCGACAACCTTTCCAA  GCAACGAGTAAACAGTCAGCGT  ATGGTGACAAGGGAGGCG  TGGAACACAGCACGAGGAA  CTCTGAGTTGGAAAGGGATCG  GCTTAGGTTGGGGAATGGG  CCGGCGACTTGATATCTCTT  CCAGTTGGGGTTGTGCTTA  GGAACACGGAAAGCCATAC  GTGCCCAGCAGAACATCAT  GCCAAAATCTTACCAGAACGAC  TGACAAGGAACTCTGGACCGT  AGAATACGGTCCCAAGTTCCTAG  CATCAAGTGATATGTGACGCTGA  ATATTCCATGAGCCCGTAGCA  CCTGTATTTCTCGTCCGTTGTC  AACCTATCGCCCTCCTCGTT  CTGGTCCATTGCTTGCTTCC  TCCAAGTGTCCTGCAAACAAC  AGCCGAACTCAAAGAATCCC  AGACAACTCCAGCATCACCG  GTTTCCCGAAGCCATCAGC  GGTTTTGGTCTTGATGCTGATT  TTCCTACCCACTGGCTCGTA  TTTTGGAGTTGAGACCTGGACC  CGTCAGCGGGGAGTGATTTA  GTTCATAGGTGCCTGTGGTTTT  CGTCCAATGGGCTTGTAGTTT  TGCGTGGATGCTGGATGA  GCGAGCTATTATGTGACCGTTT  AATGCAGGACTGAAGGACCAA  CGGAACCAATGAAGAGCCAC  ACAATTACGCACATCCATTCCT  GACTTTCTTCGAGACTCCAACG  CTGGTATGATCGGCGGTTCT  TTGAGGCACAGTGTCGCTAAG  TGGAAGTGGGACAAGGATGA  GGGAACACCAACTAGAGGGACT  CCACAAACCCCGCATACAC  AAAGCCCTTTCTTGGACACC  ATCGCAGCAACGCAAAGA  CTCCATCCAGTACAGCAACTCC  GGAAGCAGGATGTGGAGCA  AGCATTGGAGTCGTGGTCG  GAGTGGTCGTCTTCATCCCG  GTTCCGCTCACGCATCTGTT  TCTGCCTAAAAGTCCGTTCACA  GCCTGATACTTTGCGGTCGTA  ACTGGGGTTGTGACTGTGAATG  AGTTGCTTTGGTAGTGCTTTGTG  GTAGGAGATGGAAACGAACCG  AGTGACACGAGAAACACCAAGAG  TTCAAGTGCGAGCCCGACTA  CAGACTAGAATGGCGAGGAACAC  CACGAACCAGCACCACAAAC  TGACCAAATCAAGCCCAGAAT  ACACCGTGCTGCCTTACAAA  GAAGTAATGGAGACCGTTTATCGT  CCGCCCCAAACCTAAATACA  GCAGCAGTTCCAAAGTCACAGA  AATTACCGACGGGCAGGAG  GACGGGTCATTTTGATACGCT  CATGTTGTGCTCACCTCGTCTC  CGCTGTCCGCATTCACTATTT  TTTGCCGATGATTTGGTGAC  CTCCCTTCTTCGTAGTGCCTC  AAGAGCCACCACCGACGATA  GCTTCAGAGTCACGGGAACTG  GGAAAAGATGTGGGATAGATGAAG  TGTGACCAGGAAAGGGCAAG  CCTATCTGTAACGGAGTTGGAAGA  AGCGTCGAGTATGGGCTGTC  CAGATAGAGCGGGACTTATGGTT  TGTGATACTTGAGCTGTGCGACT  GAATCCCAGCCTTGTCCGTA  AGAGGGCAGAGCGATGAAAC  GCGTCTGCTCCAAATCCAA | Sl-CP-F  Sl-CP-R  Sl-Peritrophin A2-F  Sl-Peritrophin A2-R  Sl-SP-F  Sl-SP-R  Sl-SP18-F  Sl-SP18-R  Sl-SP20-F  Sl-SP20-R  Sl-SP37-F  Sl-SP37-R  Sl-5-HR1-F  Sl-5-HTR1-R  Sl-A2b-RT-F  Sl-A2b-RT-R  Sl-AC3-RT-F  Sl-AC3-RT-R  Sl-CaBPE63-1-RT-F  Sl-CaBPE3-1-RT-R  Sl-CACNB2-RT-F  Sl-CACNB2-RT-R  Sl-Calmodulin-A-RT-F  Sl-Calmodulin-A-RT-R  Sl-CaMcAMPase-RT-F  Sl-CaMcAMPase-RT-R  Sl-EGFR-RT-F  Sl-EGFR-RT-R  Sl-Eip63F-1-RT-F  Sl-Eip63F-1-RT-R  Sl-ITPR-RT-F  Sl-ITPR-RT-R  Sl-MCCSA-RT-F  Sl-MCCSA-RT-R  Sl-PLCB1-RT-F  Sl-PLCB1-RT-R  Sl-PLCB4-RT-F  Sl-PLCB4-RT-R  Sl-PLCG-A-RT-F  Sl-PLCG-RT-R  Sl-PMCA-RT-F  Sl-PMCA-RT-R  Sl-PMCA2-RT-F  Sl-PMCA2-RT-R  Sl-RyR-RT-F  Sl-RyR-RT-R  Sl-SERCA-RT-F  Sl-SERCA-RT-R  Sl-Tyramine receptor 2-RT-F  Sl-Tyramine receptor 2-RT-R  Sl-VGCCA-RT-F  Sl-VGCCA-RT-R  Sl-ASPP -RT-F  Sl-ASPP-RT-R  Sl-Caspase-4-RT-F  Sl-Caspase-4-RT-R  Sl-Collagen-RT-F  Sl-Collagen-RT-R  Sl-IRSs-RT-F  Sl-IRSs-RT-R  Sl-ITGB1-RT-F  Sl-ITGB1-RT-R  Sl-LAMA -RT-F  Sl-LAMA-RT-R  Sl-LRCH1-RT-F  Sl-LRCH1-RT-R  Sl-MEGF8-RT-F  Sl-MEGF8-RT-R  Sl-PDK1-RT-F  Sl-PDK1-RT-R  Sl-PPM1D-RT-F  Sl-PPM1D-RT-R  Sl-RnrS-RT-F  Sl-RnrS-RT-R  Sl-Sinah-RT-F  Sl-Sinah-RT-R  Sl-Triokinase-RT-F  Sl-Triokinase-RT-R  Sl-VAT1-RT-R | AGCGGTGACTTGGTAAATGAAA  CTAAGGACCTGGTTGACTGAGACT  GTGTTGTGATCGCTGTAGTGGC GGTGGTGCAAGGAACCCATT  CCAACTCAGTCTGCGGAAACA  GCCGATACCGATTAGAACCCT  GAAGCACCAATGACGAACAAA  AATCTCCACCACAAATACCCAC  CAAACGATGAATGCGAACAAG  GAATGAAACGACACCAATCACTAC  TATCGCTCTTCCTTCTGGGTC  CTTGTTCGCATTCATCGTTTG  TACATCGCTGTCTTTCCCTCAG  TGTCGAATTTACGCATCTCCA  CGAGGTGATGACAGTTGACGG  GGCATGAAGGCTTTGATTGC  AGTCCTTCAACCATTTCGTGC  CCGCTTTGCCTGTACTTTCC  GTAGTCCCAGCACCCAGTGAA  GCCCTTGTAACGCCTGTATCTT  GCTTGTGGTGTTGGACTGTGA  ACCCTTGCCACGAGATTTTAT  AGGGAGGCTTTCAGGGTATTC  AATTGACCATGCCATCACCA  GCTGGAGTCGCTGAGTGCTA  TTTCTCGTGATAGACTCGCTGAC  ACGCAGAACGCTTCCACAT  TGCCGCTACCCTCACTCATA  CGACGACCTCATCGGTGAAC  GCCCTTGTAACGCCTGTATCTT  GTTGGCTGTATGGAGGTGAATG  AACGCCTTACTGCTGGTCG  TTCATTTATGCTGTCGTTGGG  TTCTTATCGCACTTCACTTCTGG  ACCTATGAATACAACCGACGATG  TCAGTCAGGAGTTGTCCCGATA  GTTCCGTCAGGGACAGTTTGT  GCTACTATTTCTTCGGGCTTCTTAT  TTGATTCCTCGAACTACAACCC  CGTAACCGCATCCACCATT  GCTCTTCAACGAAATCAACGC  GCCGAGGAACAGACACCAGA  GCTCTTCAACGAAATCAACGC  GCCGAGGAACAGACACCAGA  CCTGCCAGCAAACAAAGATC  ACCTCCGAAGACATAACCCAC  GAACCCCTTCAATGTCCCC  GTCCATTGCCAAGACGAGAA  TCCTATTACCACCCGAACGAC  AGCCCCTTCCTCAGATCCTC  GTCAATCAAAATGGTGGCAATG  GGCAACGTGTCCAGTAAAAGC  ATTGGATTTGGGATCGTTAGG  CGGTTTAGGGGATTTGTCTGT  TCTACCTCGGCTTGGGACTG  TGCTGGTCTTTTGCCCTTAGTA  GGGGTAGTAACCGCCTGATG  TGAGACCACGAATGTCAAACCTA GCCCGACGCCAATCATATT  GCCCTTGACGGCATACTAACA  CTTTGTTTCGGACACGGTAGG  ATGCCTCAGTGGCGTATGG  GCAACTGGCATAAGATTCACG  TGAGTGGTATTTTGAGGGGTGTT  AGTATGCCAGATGCCACTTCA  GACTTGCGTGAGCCTGTTGT  TTATGCCCCAATGGATGCT  TCTGTATGTGACAACCAGTGCC  TCAGATTTGTGGGCGTTGG  GGATGGTTGCGAATACTGTCATA  GTTGGGGATTCAGCGATTG  TTCTTCTTGATAGGACCCTTGTG  ATATGAGTGGCGAAAGCATGG  AAAACGGCGAGGGTTGTCT  GTTGGTATCATCCGAAGAAGGC  ACGCAGGGTCCACAGCATT  TCAGGGATAAAGGTGGAAGGTC GAGCAAGCACTAAGACAGACGC GCCACAAAGGCAGTCCAAC |

|  |  |
| --- | --- |
|  |  |
